# *Escherichia coli* multilocus sequence type 38 from humans and broiler production represent distinct monophyletic groups

**DOI:** 10.3389/fmicb.2023.1173287

**Published:** 2023-05-02

**Authors:** Solveig Sølverød Mo, Eve Zeyl Fiskebeck, Jannice Schau Slettemeås, Karin Lagesen, Oskar Nilsson, Umaer Naseer, Silje Bakken Jørgensen, Thorunn Rafnar Thorsteinsdottir, Marianne Sunde

**Affiliations:** ^1^Section for Food Safety and Animal Health Research, Department of Animal Health, Welfare and Food Safety, Norwegian Veterinary Institute, Ås, Norway; ^2^Section for Epidemiology, Department of Animal Health, Welfare and Food Safety, Norwegian Veterinary Institute, Ås, Norway; ^3^Department of Animal Health and Antimicrobial Strategies, National Veterinary Institute (SVA), Uppsala, Sweden; ^4^Department of Infection Control and Preparedness, Norwegian Institute of Public Health, Oslo, Norway; ^5^Department of Clinical Microbiology and Infection Control, Akershus University Hospital, Lørenskog, Norway; ^6^Department for Laboratory Medicine, Vestre Viken Hospital Trust, Drammen, Norway; ^7^Institute for Experimental Pathology, University of Iceland, Keldur, Iceland

**Keywords:** *Escherichia coli*, ST38, OXA-244, comparative genomics, ESC, broilers

## Abstract

*Escherichia coli* belonging to multilocus sequence type 38 (ST38) is a well-known cause of extra-intestinal infections in humans, and are frequently associated with resistance to extended-spectrum cephalosporins (ESCs). Resistance to carbapenems, mediated by *bla*_OXA_-genes has also been reported in this ST. Recently, the European Centre for Disease Prevention and Control (ECDC) released a rapid risk assessment on the increased detection of OXA-244 producing *E. coli* ST38 in humans, requesting further knowledge to determine the source. ST38 is also one of the most common STs among ESC-resistant *E. coli* from broiler production. Our aim was to investigate the genetic characteristics and relationship between *E. coli* ST38 from broiler production and humans, and to investigate if there has been a potential spillover between these sources. A total of 288 *E. coli* ST38 genomes isolated from humans in Europe (collected 2009–2019) and from Nordic broiler production (collected 2011–2014) were analyzed. The results showed distinct monophyletic clades associated to humans and broiler production. Furthermore, there were differences in the ESC resistance genes present in *E. coli* ST38 from the two sources. The *bla*_OXA-244_ gene was not present in *E. coli* from broiler production. Our results show that ST38 from humans and broiler production belong to well-separated clades, and suggest that the increased detection of OXA-244-producing *E. coli* ST38 in humans is not associated with spillover from broiler production.

## 1. Introduction

Antimicrobial resistance (AMR) poses a serious threat to public health. Infections caused by resistant bacteria have limited treatment options, are costly to treat, and lead to increased morbidity and mortality (Dziekan et al., 2012). Worldwide, broiler production is associated with occurrence of extended-spectrum cephalosporin (ESC) – resistant *Escherichia coli* (Hiroi et al., 2012; EFSA and ECDC, 2022). This has also been the case in Nordic countries, despite negligible use of antimicrobials (Agersø et al., 2014; Mo et al., 2014; Myrenås et al., 2018). Trade of breeding animals has contributed to the wide dissemination of ESC-resistant *E. coli* in broiler production, as the same breeding animals supply the production in many countries (Agersø et al., 2014; Myrenås et al., 2018). Sequence type (ST) 38 has been shown to be predominant among ESC-resistant *E. coli* from Norwegian and Nordic broiler production (Mo et al., 2016; Myrenås et al., 2018; Buberg et al., 2021), as well as from several other European countries (Dierikx et al., 2013; Hansen et al., 2016; Seiffert et al., 2017; Pietsch et al., 2018).

*E. coli* ST38 has also been reported as a common cause of extra-intestinal infections in humans, particularly urinary tract infections (Chattaway et al., 2014). In addition, these isolates are often ESC resistant, and have been associated with *bla*_OXA-48_- (Potron et al., 2013; Turton et al., 2016) and *bla*_OXA-244_- (ECDC, 2021) mediated carbapenem resistance. A recent increase of *E. coli* ST38 with *bla*_OXA-244_ in the European Union/European Economic Area (EU/EEA), followed by an outbreak of health-care associated infections in Norway prompted the European Centre for Disease Prevention and Control (ECDC) to perform two rapid risk assessments on the increased detection of OXA-244 producing *E. coli* ST38 in humans (2020 and 2021) (ECDC, 2020, 2021). Currently, the source and transmission route of OXA-244-producing *E. coli* ST38 in EU/EEA is unknown, and further knowledge is required to implement adequate control measures. Transmission via food, environment or direct contact with animals is possible, but more data are required to elucidate the potential role of these sources (ECDC, 2021). In general, in-depth studies of ST38 from humans and their possible link to animal origin should be prioritized to explore the molecular epidemiology of this important pathogen.

In this study, we have used whole genome sequencing (WGS) to compare *E. coli* ST38 originating from broiler production and humans. Our aim was to investigate the genetic characteristics and relationship between *E. coli* ST38 from these two sources, and to investigate if there has been a potential spillover between them.

## 2. Materials and methods

### 2.1. Inclusion of genomes and isolates

A total of 288 *E. coli* ST38 genomes were included in this study by retrieving genomes from public sources, and by sequencing in-house strain collections. A complete overview of all included sequence data is presented in Supplementary Table 1. Sequence data from 153 *E. coli* ST38 isolates of human origin were retrieved from public repositories, namely the European Nucleotide Archive (ENA, *n* = 136), and from project partners in Norway (Akershus University Hospital, Vestre Viken Hospital Trust and the Norwegian Institute of Public Health, for these combined *n* = 17) (Jørgensen et al., 2017; Espenhain et al., 2018). The following inclusion criteria had to be fulfilled to include an ST38 genome of human origin in the study; (i) isolate confirmed as *E. coli* ST38 in Enterobase or publication, (ii) Illumina sequencing data available from ENA or project partner, (iii) isolates originating from European countries where *E. coli* ST38 have been reported from broiler production, and (iv) isolates originating from patients or healthy carriers, not necessarily ESC resistant. Included genomes originated from human isolates collected between 2009 and 2019.

We also included sequence data from 135 *E. coli* ST38 originating from broiler production. Sequence data from 73 *E. coli* ST38 isolates from broiler production were available from previous studies (Berg et al., 2017; Buberg et al., 2021). Furthermore, a selection of ESC-resistant *E. coli* from Norwegian broiler production (*n* = 67) were analyzed with MLVA (Mo et al., 2016) to identify isolates displaying an ST38-associated MLVA profile (Supplementary Table 2; Myrenås et al., 2018). Of these, 63 displayed an ST38-associated MLVA-profile and were sequenced as part of the present study (Supplementary Table 2). Included genomes from broiler production originated from isolates collected between 2011 and 2014 in three Nordic countries; Norway, Sweden and Iceland. From previous studies, all isolates from broiler production were known to carry the *bla*_CMY-2_ gene encoding ESC resistance (Mo et al., 2014; NORM/NORM-VET, 2015; Myrenås et al., 2018).

### 2.2. DNA extraction

For the isolates sequenced in this study, DNA was extracted manually from fresh colonies using the QIAmp DNA mini kit (Qiagen, Hilden, Germany) or automated on a QIAsymphony (Qiagen) using the DSP DNA Mini Kit (Qiagen). DNA concentration was determined on a Qubit® (Thermo Fisher Scientific) using the dsDNA Broad Range Assay Kit and the DNA purity was measured on a NanoDrop spectrophotometer (Thermo Fisher Scientific). For one isolate (2012-01-1292), DNA for long-read sequencing was extracted as described previously (Mo et al., 2020).

### 2.3. Whole genome sequencing

Samples were prepared with a Nextera library preparation kit (Illumina) and sequenced on a HiSeq3/4000 or a NextSeq 500 Illumina platform obtaining 150 bp paired-end reads. A complete overview of library preparation kits and sequencing platforms used for the different isolates is provided in Supplementary Table 1. Isolate 2012-01-1,292 was also sequenced using Pacific Biosciences (PacBio) long-read sequencing as previously described (Mo et al., 2020).

### 2.4. Pre-processing and *in silico* characterization of sequence data

*In silico* characterization of all genomes was done using the plasmid assembly, gene identification and annotation pipeline Ellipsis v 0.5.2 with raw Illumina reads as input (Kaspersen, 2021). Briefly, the pipeline assessed read quality with FastQC^1^ and summarized it with MultiQC (Ewels et al., 2016). Reads were trimmed by TrimGalore v 0.6.4 (Krueger, 2019), and assembled by Unicycler v 0.4.8 (Wick et al., 2017) using default settings. ResFinder (database downloaded 11.02.2020) (Zankari et al., 2012) and PlasmidFinder (database downloaded 06.03.2020) (Carattoli et al., 2014) were used to identify AMR genes and plasmid replicons in the assembled genomes. Assembly quality was assessed with QUAST v 5.0.2 (Gurevich et al., 2013), and STs were predicted with MLST^2^ using the Achtman scheme (Wirth et al., 2006; Jolley and Maiden, 2010).

Long-reads from isolate 2012-01-1292 were demultiplexed by the SMRT Link barcoding pipeline (v6.0.0.47841, SMRT Link Analysis Services and GUI v6.0.0.47836) with 40 as minimum barcode score. Thereafter, the short and long reads from this isolate were assembled using Unicycler v 0.4.8 with default settings for hybrid assembly (Wick et al., 2017).

### 2.5. Phylogeny

A core SNP based phylogenetic analysis was performed with the ALPPACA pipeline (A tooL for Prokaryotic Phylogeny and Clustering Analysis) (Kaspersen and Fiskebeck, 2022) using the mapping-track to investigate the genetic relatedness within the ST38 group. A core SNP alignment was reconstructed using Snippy v 4.6.0.^3^ The hybrid assembly of isolate 2012-01-1292, originating from retail chicken meat, was used as reference for the SNP calling. The coverage of the core genome included in Snippy was calculated as the minimum proportion of the included genomes that aligned to the reference genome. Recombinant regions were identified with Gubbins v 3.2.0 (Croucher et al., 2015), using the GTRGAMMA model and RaxML as initial tree builder. Putative recombinant regions were masked using maskrc-svg v 0.5^4^ before filtering using snp-sites v 2.5.1 (Page et al., 2016). A maximum likelihood (ML) phylogeny was generated using IQ-TREE v 2.2.0.3 (Minh et al., 2020). ModelFinder (Kalyaanamoorthy et al., 2017) was used to determine the most suitable evolutionary model, and UFBoot2 to evaluate branch support (Hoang et al., 2018). An *E. coli* ST115 genome (accession no ERR4706315) originating from retail chicken meat was included as an outlier in the phylogeny. Snp-dists v 0.8.2^5^ was used to calculate the pairwise SNP distances between genomes using the recombinant-filtered alignment. The phylogenetic trees were visualized and annotated using the ggtree package v 3.0.4 (Yu et al., 2017, 2018; Yu, 2020) in R v 4.1.1 (R Core Team, 2021).

### 2.6. Statistics

Chi-square tests were performed in R v 4.1.1 (R Core Team, 2021).

## 3. Results

### 3.1. *In silico* characterization

An overview of the results from the *in silico* characterization of genomes is presented in Supplementary Tables 3, 4. Of the 63 broiler isolates sequenced in this study, 62 belonged to ST38. Thus, 135 *E. coli* ST38 from broiler production were included in addition to the 153 genomes originating from human isolates (Supplementary Tables 1, 2).

### 3.2. Phylogenetic, ESC resistance gene, and plasmid diversity

**Figure 1:**
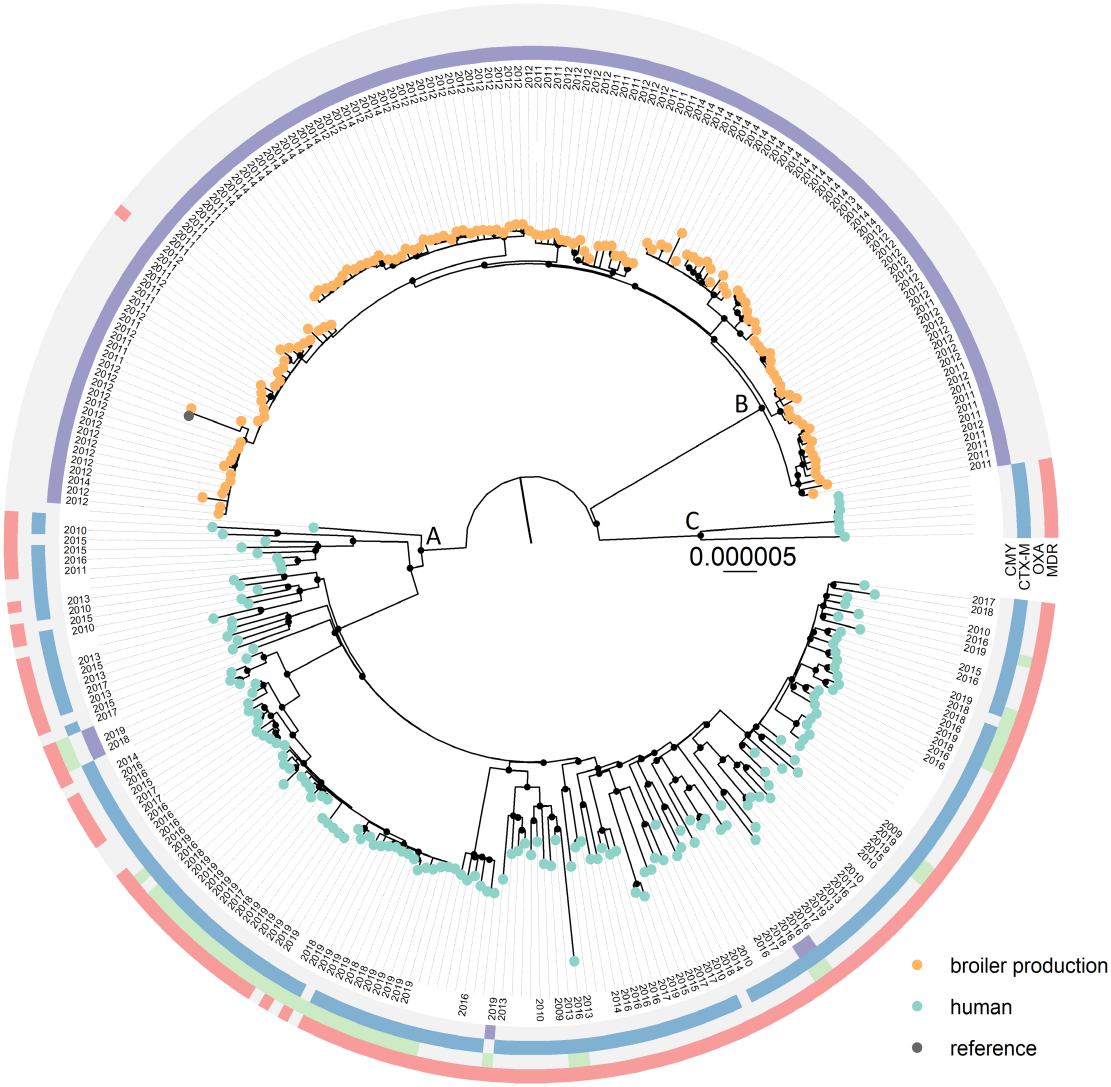
Maximum likelihood core genome SNP tree of 288 *Escherichia coli* multilocus sequence type 38 originating from humans (*n* = 153) and broiler production (*n* = 135). Presence (CMY = purple, CTX-M = blue, OXA = green, MDR = red) and absence (light grey) of relevant antimicrobial resistance genes and multidrug resistance are displayed in the outer circles. Only *bla*_OXA-48_, *bla*_OXA-181_, and *bla*_OXA-244_ are included in the OXA-group. Both *bla*_CMY-2_ and *bla*_CMY-16_ are included in the CMY-group, and *bla*_CTX-M-1_, _−3_, _−9_, _−14_, _−14b_, _−15_, and _−27_ are included in the CTX-M group. Black circles on nodes represent accepted bootstrap values (>95%). Color on tip-points indicate origin of the ST38 genomes. The tree is rooted using an outgroup (*E. coli* ST115).

The core genome covered 81.0% of the reference genome before masking recombinant regions. The phylogenetic analysis revealed that *E. coli* ST38 from humans and broiler production belong to distinct monophyletic clades, namely Clades A and B. A few ST38 genomes of human origin belonged to Clade C, a sister-clade to Clades A and B (Figure 1, Supplementary Figure 1 for rectangular view). There was a minimum of 177 SNP differences between Clades A and B, 293 SNPs between Clades B and C, and 205 SNPs between Clades A and C. When comparing genomes within each of the clades, ST38 of human origin differed by 0–341 SNPs in A and 0–211 SNPs in C, while ST38 from broiler production differed by 0–79 SNPs (B).

#### 3.2.1. Acquired AMR genes and plasmid replicons

The *bla*_CMY-2_ gene was present in 100% of the ST38 genomes originating from broiler production (Supplementary Table S3). In addition to *bla*_CMY-2_, some harbored *bla*_TEM-1b_ (*n* = 29), *sul2* (*n* = 2), *dfrA1* (*n* = 1) and *aadA1* (*n* = 1). Only a single *E. coli* ST38 from broiler production was predicted to be multidrug resistant (MDR, i.e., resistant to three or more antimicrobial classes, Figure 1), and none carried *bla*_OXA_-genes (Figure 1, Supplementary Table S3).

Genes encoding ESC resistance were identified in 146 (95.4%) of the 153 ST38 genomes originating from humans, with *bla*_CTX-M-27_ (*n* = 45) and *bla*_CTX-M-15_ (*n* = 44) being the most common (Figure 1, Supplementary Table S3). *bla*_OXA-244_ was present in 43 of the human ST38 genomes. In 41 of these, *bla*_OXA-244_ was present in combination with a gene encoding ESC resistance, mainly *bla*_CTX-M-27_ (*n* = 29). In addition, *bla*_CMY-2_ was present in four genomes, and *bla*_CMY-16_, *bla*_OXA-48_ and *bla*_OXA-181_ were present in two genomes each. There was a significant difference between the occurrence of predicted MDR among ST38 from broiler production and humans (*p* < 0.001) as 140 (91.5%) of the *E. coli* ST38 of human origin were predicted to be MDR compared to one (0.7%) of broiler origin (Figure 1). A complete overview of additional acquired AMR genes is presented in Supplementary Table S3.

The four *E. coli* genomes of human origin carrying *bla*_CMY-2_ only harbored IncF replicons, while the majority originating from broiler production harbored both IncB/O/K/Z and IncF replicons. A complete overview of plasmid replicons detected in the included genomes is presented in Supplementary Table S4. Raw sequencing reads from isolates sequenced in the present study are available from ENA (accession numbers PRJEB52719, PRJEB52740, PRJEB52781 and PRJEB52832).

## 4. Discussion

The results from our phylogenetic analysis showed that the genomes clustered into distinct monophyletic groups, with all ST38 from broiler production in a clade separate from those originating from humans. The within-group SNP distances indicated that the ST38 genomes from broiler production were more homogenous than those originating from humans. The maximum within-group SNP distances were 341 and 211 in Clades A and C, respectively, and 79 in Clade B. However, our dataset may have some limitations, and comparison and interpretation of the data should be performed with some caution. Isolates from humans were collected over a period of 10 years, while isolates from broiler production were collected during a four-year period. Also, the human isolates originated from several European countries, while isolates from broiler production were from Nordic countries and had a common ancestry (Myrenås et al., 2018). All ST38 from broiler production were ESC resistant and isolated using selective methods, while this was not the case for all isolates of human origin. Additionally, isolates from humans originated from both healthy humans and patients, while all ST38 from broiler production were from healthy animals. In broiler production, a high level of biosecurity is implemented to prevent the introduction of infectious agents, possibly preventing the introduction of several different *E. coli* ST38 strains. However, this needs to be further investigated. These factors may have influenced the heterogeneity observed in *E. coli* ST38 of human origin compared to ST38 from broiler production.

Our results show two distinct sub-populations within the same ST. The presence of sub-populations has also been described for other STs, such as ST131, associated with extra-intestinal *E. coli* infections in humans (Stoesser et al., 2016), where clinical isolates often belong to the sub-lineage H30Rx (Price et al., 2013). The occurrence of different sub-populations in ST38 may be due to adaptation to different hosts, namely humans and broilers, over time.

We detected large variations in the presence of acquired AMR genes. *bla*_CMY-2_ was the only ESC-encoding gene detected in ST38 from broiler production. In ST38 from humans, a diverse set of ESC resistance genes was present, with genes in the *bla*_CTX-M_-group most commonly detected. The *bla*_CMY-2_ gene was present in combination with IncF replicons in ST38 genomes of human origin. We have previously shown that *bla*_CMY-2_ is located on IncK or IncI1 plasmids in *E. coli* from broiler production, and that ST38 is associated with *bla*_CMY-2_/IncK plasmids (Mo et al., 2016). Thus, there is no evidence of common *bla*_CMY-2_-carrying plasmids in ST38 from humans and broiler production.

*E. coli* ST38 carrying *bla*_OXA-48_ or *bla*_OXA-244_ encoding carbapenem resistance has emerged in the human clinical setting (Potron et al., 2013; Turton et al., 2016; ECDC, 2020, 2021). Recently, the occurrence of *E. coli* ST38 with *bla*_OXA-244_ has increased in European countries, including Norway. The reason for this increasing number of cases is unknown (ECDC, 2021). We included 43 OXA-244 producing ST38 of human origin in our analyses. All of these clustered together with the rest of the human ST38 genomes, displaying a considerable number of SNPs to ST38 genomes from broiler production. None of the ST38 genomes from broiler production carried any *bla*_OXA_-genes. Based on our results, it seems that the increased detection of OXA-244-producing *E. coli* ST38 in EU/EEA is not due to spillover from broiler production. The *E. coli* ST38 genomes included in our study are divided into sub-groups consisting of isolates from humans and broiler production, respectively. This may suggest host adaptation of isolates belonging to the distinct sub-groups. There is no indication of transmission of *E. coli* ST38 between the human and broiler reservoirs in our data. However, our dataset have some limitations as discussed above. Thus, additional studies with detailed and extensive sample- and data collection is required in order to determine whether host specificity is present, or if and to what degree transmission between reservoirs may occur.

In conclusion, our results show the presence of distinct sub-groups within *E. coli* ST38, possibly adapted to separate hosts. To our best knowledge, this is the first in-depth characterization of a large number of *E. coli* ST38 from humans and broiler production using WGS.

## Data availability statement

The datasets presented in this study can be found in online repositories. The names of the repository/repositories and accession number(s) can be found in the article/Supplementary material.

## Author contributions

MS, SM, and SJ contributed to the conception and design of the present study. SM, JS, ON, UN, SJ, TT, and MS contributed to the acquisition of isolates and sequencing data. SM and JS performed the laboratory work. SM, EF, KL, and JS performed the *in silico* analyses. SM wrote the first draft of the manuscript. All authors contributed to manuscript revision, read, and approved the submitted version.

## Funding

The study was funded by the Research Council of Norway, grant no. 250212, and the Norwegian Veterinary Institute.

## Conflict of interest

The authors declare that the research was conducted in the absence of any commercial or financial relationships that could be construed as a potential conflict of interest.

## Publisher’s note

All claims expressed in this article are solely those of the authors and do not necessarily represent those of their affiliated organizations, or those of the publisher, the editors and the reviewers. Any product that may be evaluated in this article, or claim that may be made by its manufacturer, is not guaranteed or endorsed by the publisher.

## Footnotes

^1^ http://www.bioinformatics.babraham.ac.uk/projects/fastqc/

^2^https://github.com/tseemann/mlst, https://pubmlst.org/

^3^ https://github.com/tseemann/snippy

^4^ https://github.com/kwongj/maskrc-svg

^5^ https://github.com/tseemann/snp-dists
