# Supplementary material for: Escherichia coli multilocus sequence type 38 from humans and broiler production represent distinct monophyletic groups
Source: Front Microbiol. 2023 May 2;14:1173287. doi: 10.3389/fmicb.2023.1173287 (PMC10231635; doi:10.3389/fmicb.2023.1173287)

Supplementary Material

# Supplementary Tables

**Supplementary Table 1**. Complete overview of all 288 *Escherichia coli* ST38 isolates included in the present study.

See datasheet (excel).

**Supplementary Table 2**. Overview of extended-spectrum cephalosporin-resistant *Escherichia coli* from broiler production subjected to whole genome sequencing in this study.

| Source | Sample material | Year | Country | Study/monitoring programme | Number of isolates | MLVA to detect ST38-associated MLVA type | Number of isolates included in WGS | Number of isolates confirmed as ST38 |
| --- | --- | --- | --- | --- | --- | --- | --- | --- |
| Chicken meat | Meat from retail | 2014 | Iceland | Myrenås et al 2018 | 5 | No | 5 | 5 |
| Broilers | Boot swabs | 2011 | Norway | NORM-VET 2011 | 31 | No | 31 | 31 |
|  | Caecal samples | 2014 | Norway | NORM-VET 2014 | 44 | Yes | 14 | 13 |
|  | Caecal samples | 2016 | Norway | NORM-VET 2016 | 3 | Yes | 0 | 0 |
|  | Caecal samples | 2011 | Sweden | Myrenås et al 2018 | 4 | No | 4 | 4 |
|  | Caecal samples | 2014 | Iceland | Myrenås et al 2018 | 1 | No | 1 | 1 |
| Parent flocks | Boot swabs | 2012 | Norway | NORM-VET 2012 | 7 | Yes | 6 | 6 |
|  | Boot swabs | 2014 | Norway | Mo et al 2016 | 4 | Yes | 2 | 2 |
| Total |  |  |  |  | 99 |  | 63 | 62 |

**Supplementary Table 3.** Occurrence of antimicrobial resistance genes in 288 *Escherichia coli* isolates originating from humans and broiler production.

| Gene | Broiler production | Human | Total |
| --- | --- | --- | --- |
| aac(3)-IIa |  | 16 | 16 |
| aac(3)-IId |  | 13 | 13 |
| aac(6')-Ib-cr |  | 17 | 17 |
| aac(6')-Ib-Hangzhou |  | 1 | 1 |
| aadA1 | 1 | 64 | 65 |
| aadA2 |  | 8 | 8 |
| aadA5 |  | 42 | 42 |
| aadA8b |  | 1 | 1 |
| aph(3')-Ia |  | 21 | 21 |
| aph(3'')-Ib |  | 116 | 116 |
| aph(6)-Id |  | 116 | 116 |
| bla_CMY-2_ | 135 | 4 | 139 |
| bla_CMY-16_ |  | 2 | 2 |
| bla_CTX-M-1_ |  | 1 | 1 |
| bla_CTX-M-3_ |  | 2 | 2 |
| bla_CTX-M-14_ |  | 18 | 18 |
| bla_CTX-M-14b_ |  | 33 | 33 |
| bla_CTX-M-15_ |  | 44 | 44 |
| bla_CTX-M-27_ |  | 45 | 45 |
| bla_DHA-1_ |  | 1 | 1 |
| bla_OXA-1_ |  | 16 | 16 |
| bla_OXA-48_ |  | 2 | 2 |
| bla_OXA-181_ |  | 2 | 2 |
| bla_OXA-244_ |  | 43 | 43 |
| bla_TEM-1B_ | 29 | 73 | 102 |
| bla_TEM-1C_ |  | 2 | 2 |
| bla_TEM-35_ |  | 3 | 3 |
| bla_TEM-210_ |  | 1 | 1 |
| catA1 |  | 32 | 32 |
| catB3 |  | 16 | 16 |
| catB8 |  | 1 | 1 |
| dfrA1 | 1 | 63 | 64 |
| dfrA5 |  | 3 | 3 |
| dfrA7 |  | 3 | 3 |
| dfrA8 |  | 2 | 2 |
| dfrA12 |  | 9 | 9 |
| dfrA14 |  | 9 | 9 |
| dfrA17 |  | 43 | 43 |
| erm(B) |  | 9 | 9 |
| floR |  | 3 | 3 |
| mph(A) |  | 61 | 61 |
| qnrB4 |  | 1 | 1 |
| qnrS1 |  | 10 | 10 |
| sul1 |  | 57 | 57 |
| sul2 | 2 | 120 | 122 |
| tet(A) |  | 63 | 63 |
| tet(B) |  | 7 | 7 |
| tet(D) |  | 39 | 39 |

**Supplementary Table 4.** Occurrence of plasmid replicons in 288 *Escherichia coli* isolates originating from humans and broiler production.

| Replicon | Broiler production | Human |
| --- | --- | --- |
| IncB/O/K/Z | 130 | 5 |
| IncFIA/FIB/FIC/FII | 131 | 129 |
| IncHI1B/HI2/HI2A | 8 |  |
| IncI1/I2 | 43 | 18 |
| IncM |  | 2 |
| IncN |  | 1 |
| IncQ1 |  | 23 |
| IncX1/X3/X4 | 3 | 9 |
| IncY | 6 | 13 |

**Supplementary Figure 1.** Maximum likelihood core genome SNP tree of 288 *Escherichia coli* multilocus sequence type 38 originating from humans (n=153) and broiler production (n=135). Presence (CMY = purple, CTX-M = blue, OXA = green, MDR = red) and absence (light grey) of relevant antimicrobial resistance genes and multidrug resistance are displayed in the outer circles. Only *bla*_OXA-48_, *bla*_OXA-181_ and *bla*_OXA-244_ are included in the OXA-group. Both *bla*_CMY-2_ and *bla*_CMY-16_ are included in the CMY-group, and *bla*_CTX-M-1_, _-3_, _-9_, _-14_, _-14b_, _-15_ and _-27_ are included in the CTX-M group. Black circles on nodes represent accepted bootstrap values (>95%). Colour on tip-points indicate origin of the ST38 genomes. The tree is rooted using an outgroup (*E. coli* ST115). This tree corresponds to Figure 1, but in a rectangular rather than circular view.


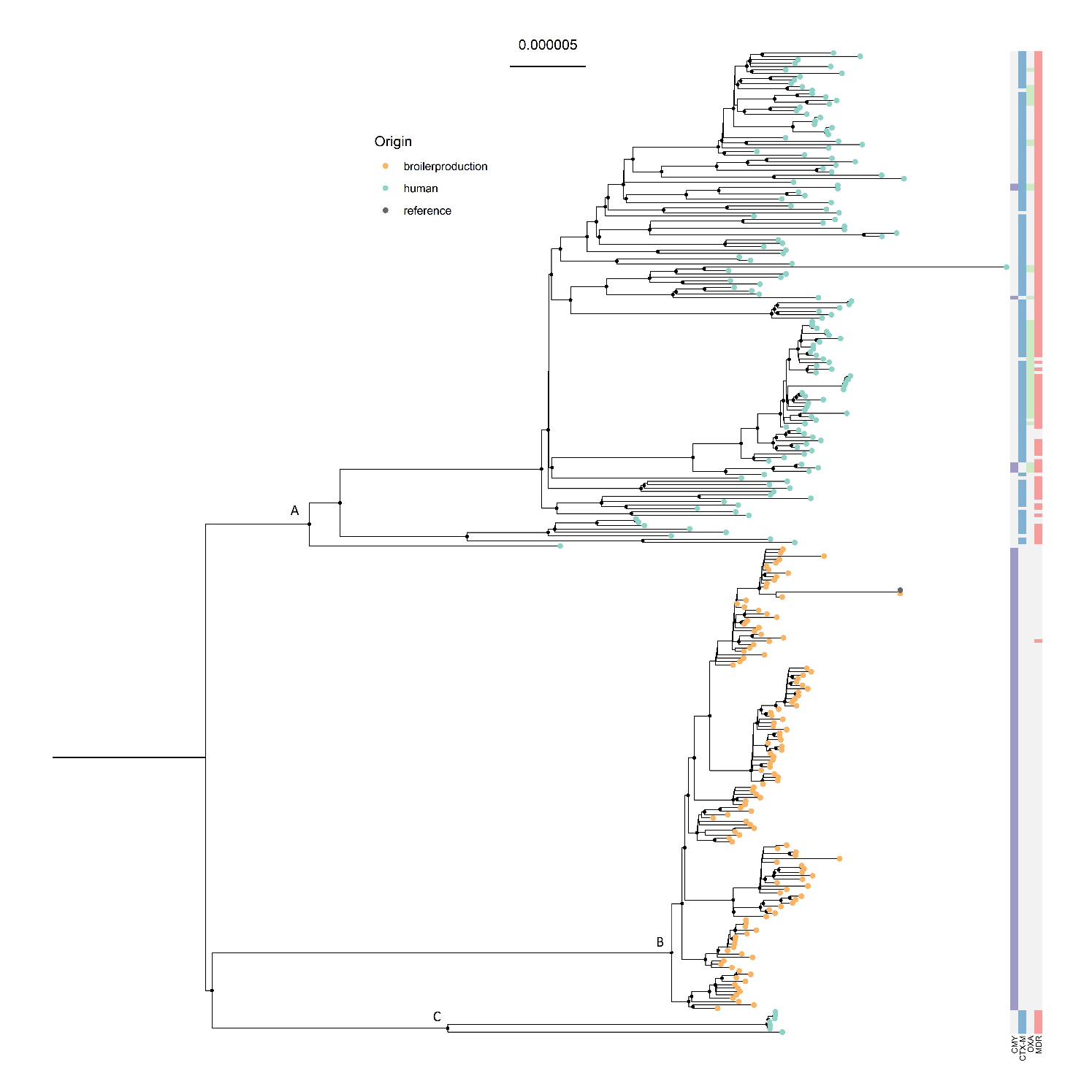

Supplement: Supplementary file 1 [file Data_Sheet_1.docx]
