# Supplementary figures and images for: Escherichia coli multilocus sequence type 38 from humans and broiler production represent distinct monophyletic groups
Source: Front Microbiol. 2023 May 2;14:1173287. doi: 10.3389/fmicb.2023.1173287 (PMC10231635; doi:10.3389/fmicb.2023.1173287)

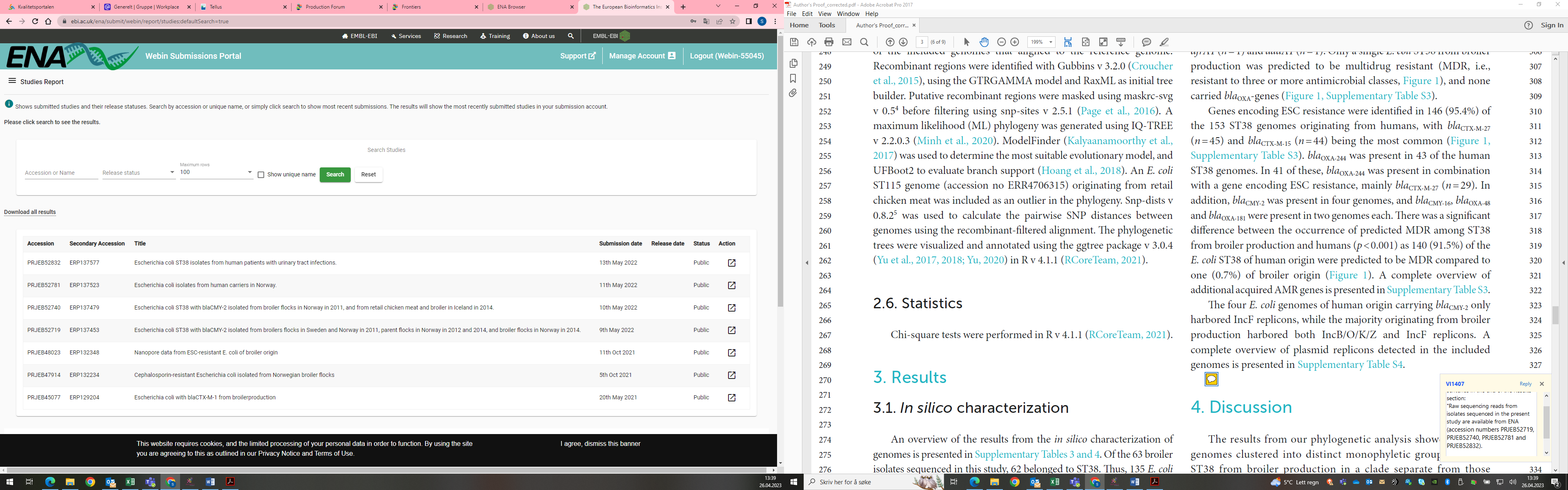

Supplement: Supplementary file 2 [file Table_1.docx]
